# Editorial: Enhancing allele mining for crop improvement amid the emerging challenge of climate change

**DOI:** 10.3389/fpls.2023.1197086

**Published:** 2023-05-26

**Authors:** Pasquale Tripodi, Narendra Kumar Singh, Michael Abberton, Amol N. Nankar

**Affiliations:** ^1^ Research Centre for Vegetable and Ornamental Crops, Council for Agricultural Research and Economics (CREA), Pontecagnano Faiano, Italy; ^2^ Department of Genetics & Plant Breeding, G. B. Pant University of Agriculture & Technology, Uttarakhand, Pantnagar, India; ^3^ Genetic Resources Center, International Institute of Tropical Agriculture (IITA), Ibadan, Nigeria; ^4^ Department of Vegetable Breeding, Center of Plant Systems Biology and Biotechnology (CPSBB), Plovdiv, Bulgaria

**Keywords:** climate resilient crops, biotic and abiotic stress, genome wide association mapping, whole genome sequencing, CRISPR-Cas, mutants

Climate change is one of the major challenges that agriculture will deal with in the upcoming years. Both unpredictable weather events and occurrence of biotic and abiotic stresses disrupt growing cycles of crops across different geographic regions, threatening food production, and leading to significant economic losses. A more effective use of plant genetic resources and innovative approaches in crop research are therefore fundamental to ensure global food security. To that end, dissecting the allelic divergence of crops and establishing breeding programs for the introduction of novel alleles may help to manage biotic and environmental stresses minimizing the losses caused by fluctuating weather events. This Research Topic collected six original research papers and one review article focusing on cereals (rice, barley, maize) and legume (peanut) discussing applications and integration of inter-disciplinary approaches for genetic movement and development of climate resilient crops (**Figure 1**).

**Figure 1:**
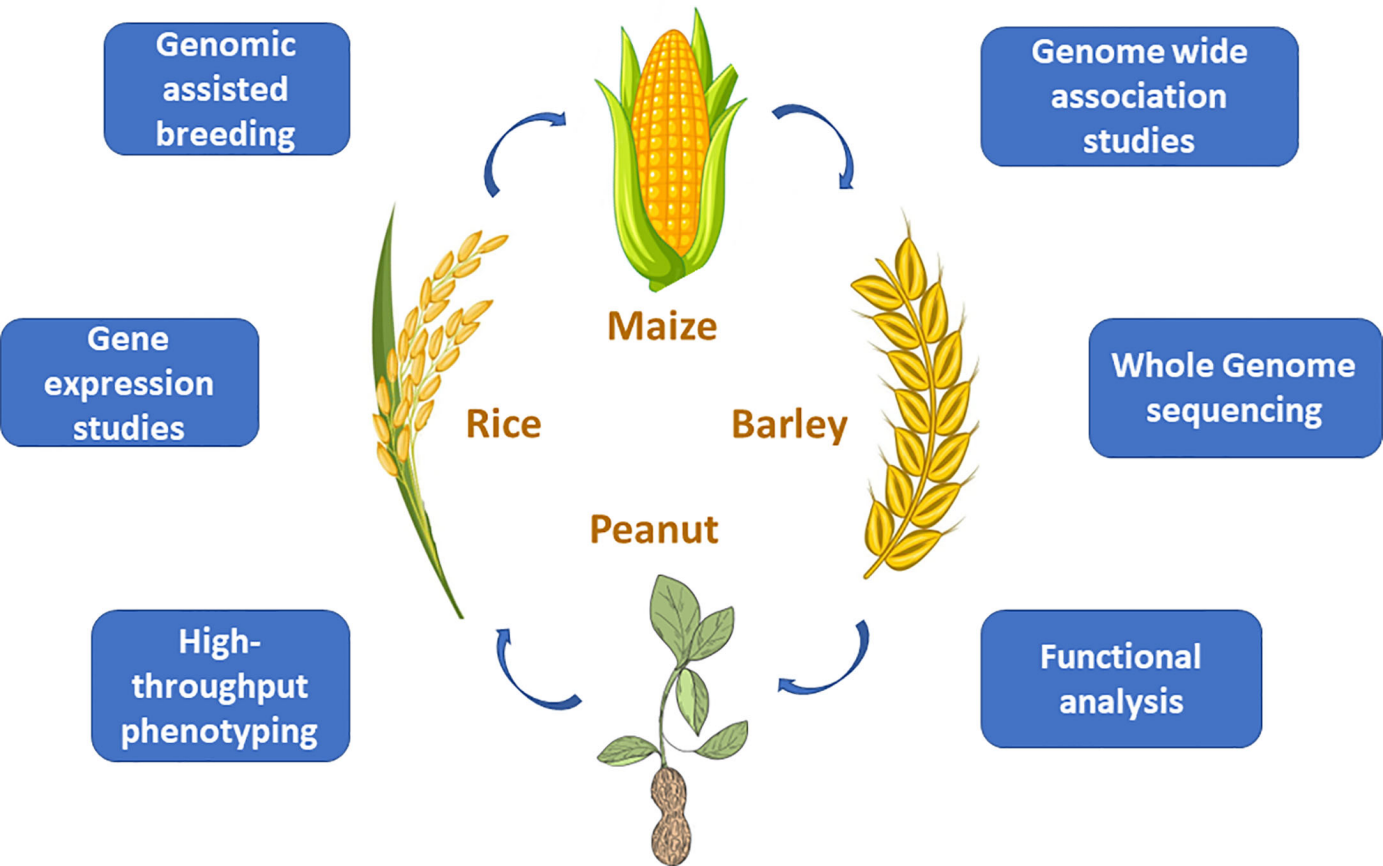
Target crops and main strategies summarized in the research topic for allele mining and crop improvement in the context of climate changes.

Genome wide association analysis (GWAS) was performed in rice (*Oryza sativa* L.) to identify alleles underlying the response to low nitrogen growth condition (Lv et al.). The authors, essaying a very diverse accessions panel built from the Rice 3K Project with over one hundred and ten thousand SNPs detected 56 single-nucleotide polymorphisms (SNPs) significantly associated to plant development, leaf length and tiller number. Associations underlined several important candidate genes including a *MYB61* transcriptional factor directly involved in cellulosic biomass production and N utilization, *MOC2* regulating growth rate, and *OsOAT* playing a key floral development and seed setting. Finally, the unknown *LOC_Os12g41090* which fall close to *OsCPK12* involved in low nitrogen responses. Through haplotype analysis the role of these candidate genes for improving nitrogen water use efficiency was demonstrated, thus shedding light into the genetic control of low-nitrogen-induced growth response. In rice, another study focused on the investigation of the role of the extra-large G protein (*XLG*) for plant growth and panicle development, as well as tolerance to abiotic stresses (Biswal et al.). Authors created novel mutants for the *XLG* gene region which significantly reduced the panicle length, number of panicles and number of grains. Functional analysis by CRISPR/CAS highlighted several frameshift mutations within 26 *OsXLG* alleles responsible for the variation of plant architecture and seed size. Furthermore, loss-of-function alleles were identified to confer salinity tolerance and hypersensitivity to pathogens.

Investigation of the genetic basis of grain size was also performed through whole genome resequencing of 20 *O. sativa* javanica and *O. sativa* indica accessions by Long et al.. The identification of genetic variants and the detection of selective sweeps in the two types allowed to identify over 100 thousand SNPs which affected about 4,852 genes specific in javanica and not present in any indica types. Through gene ontology and KEGG enrichment analysis genes involved in nucleic acid polymerase activities were overrepresented in javanica, thus putatively responsible of the higher grain size. Haplotype analysis allowed the identification of three candidate genes including *TGW2* a main gene determining grain width and weight in rice. By confirming previous finding, the study provides novel genomic resources for allele mining in rice cultivars.

Another approach in javanica rice exploited allele pyramiding in near isogenic lines combining the *starch branching enzyme 3* (*SBE3*) and *granule-bound starch synthase 1* (*GBSS1*): two alleles responsible for the high amylose and resistant starch content (Shim et al.). The associations between *SBE3* and *GBSS1* sequence variation and starch related traits highlighted changes in morphology of grains and starch physico-chemical composition. Profiling the expression levels of the two genes in panicle and investigation of their role in the variation of other starch-related genes were performed. The study shed light on the interaction between genes involved in grain quality thus suggesting strategy to develop improved cultivars.

In barley (*Hordeum vulgare*), Sallam et al. reported the identification of candidate genes for tolerance to low temperature in the germplasm collection held at the Vavilov Institute. After the first screening of over 2,000 accessions, a selected panel of 267 genotypes were analyzed with the 9K barley SNP chip and tested across six cold-weather environments. GWAS allowed detection of 12 associations on six chromosomes for genes encoding for different types of proteins playing an important role in cold acclimation and tolerance to freezing conditions. Haplotype analysis highlighted several alleles favorable for winter stress cultivation, allowing furthermore to identify a subset of high performing accessions to be used for winter barley breeding programs.

Beyond cereals, efforts toward the establishment of novel genomic resources for virginia-type peanut (*Arachis hypogaea* subsp. *hypogaea*) have been described (Newman et al.). Authors firstly developed a *de novo* genome of the ‘Bailey II’ cultivar using a combination of Pac-Bio SMRT long-read sequencing and optical mapping, then resequenced by Illumina short-read sequencing, 66 virginia-type peanut lines part of the breeding program carried out by North Carolina State University. The new assembled genome resulted in greater contiguity with respect to previously developed peanut genomes, thus providing an improved reference for the community. Genome sequencing of the breeding panel allowed the development over 1 million SNPs markers used for precise detection of introgressions from wild relatives conferring resistances to pathogens. In order to provide different genetic and genomic resources to boost assisted breeding, both PCR Allele Competitive Extension assays, and high-quality SNPs were defined and validated. The study provides a valuable resource for breeding and development of superior peanut cultivars.

Finally, in the review article Sheoran et al. discussed classical and advanced breeding strategies utilized in comprehending drought stress tolerance mechanisms and the development of resistant cultivars in maize (*Zea mays* L). The review summarized several aspects including QTL mapping strategies, GWAS and omics approaches, high-throughput and precision phenotyping, epigenetic modifications, and genome editing.

Overall, the Research Topic gives a comprehensive view of strategies and results achieved for mining the genetic variation to be used for crop improvement in the context of climate changes.

## Author contributions

All authors listed have made a substantial, direct, and intellectual contribution to the work and approved it for publication.

